# Motor Preparation and Execution for Performance Difficulty: Centroparietal Beta Activation during the Effort Expenditure for Rewards Task as a Function of Motivation

**DOI:** 10.3390/brainsci11111442

**Published:** 2021-10-29

**Authors:** Ricardo A. Wilhelm, A. Hunter Threadgill, Philip A. Gable

**Affiliations:** 1 Department of Psychology, The University of Alabama, Tuscaloosa, AL 35487, USA; rawilhelm@crimson.ua.edu; 2 Departments of Biomedical Sciences and Psychology, Florida State University, Tallahassee, FL 32306, USA; ahunterthreadgill@gmail.com; 3 Department of Psychological and Brain Sciences, University of Delaware, Newark, DE 19716, USA

**Keywords:** beta activation, motor preparation, motivation

## Abstract

Debate exists as to the effects of anxiety in performance-based studies. However, no studies have examined the influence of motivation both in preparation of a motor movement and during movement performance. The present study measured beta activation in preparation for and during execution of the effort expenditure for rewards task (EEfRT), a button-pressing task consisting of easy and hard trials. Results indicated that motor preparation (i.e., reduced beta activation) was greater in preparation for hard trials than for easy trials. Additionally, motor preparation decreased (i.e., beta activation increased) over the course of hard trial execution. These results suggest that motor preparation is enhanced prior to more challenging tasks but that motor preparation declines as participants become closer to completing their goal in each challenging trial. These results provide insight into how beta activation facilitates effort expenditure for motor tasks varying in difficulty and motivation. The impact of these results on models of anxiety and performance is discussed.

## 1. Introduction

Physical movement is possible through the sequential process of cortical motor-action preparation that precedes execution of the prepared action [1]. Past research has examined this preparatory process to contexts varying in motivational strength (i.e., rewards vs. no rewards) [2,3] but has not thoroughly examined how expectations of difficulty may impact this process. That is, how perceived expectancies about the task difficulty influence neural motor preparation, a neural correlate of motivational intensity [2,4]. From an evolutionary perspective, difficult tasks or situations that demand more effort are often accompanied by unpleasant aversive sensations that may undermine an individual’s drive to persist in some activity or attain resources [5]. Recent research, however, suggests that situations demanding more effort (i.e., more difficult situations) may not necessarily always attenuate motivational impetus to maintain behavior and may even promote sustained engagement in the activity [6]. Some recent evidence suggests more engagement for a difficult activity may arise from motivation for a more difficult task or challenge, as this may attenuate the negative effects of difficult activities (i.e., tension/anxiety) [7,8]. Therefore, neural motor-action preparation may be practical for examining immediate activation of motivational impetus to engage in difficult activities.

### 1.1. Beta Activity and Motor Functioning

Beta band frequency activity (13–30 Hz) decreases over the motor cortex prior to physical movement [1]. For example, Yuan et al. [9] showed decreased beta activity over motor cortical areas prior to hand movements. Decreased beta activity is also associated with enhanced performance. For instance, van Wijk et al. [10] asked participants to squeeze a bulb in each hand while waiting for a cue prompting an increase in squeeze force for one hand. Decreased beta activity prior to the increase in force was related to faster squeezing with the correct hand. Consequently, decreased beta activity is also associated with a greater ability to engage in regulatory control of actions and planned motor movements [11]. Overall, this indicates decreased beta activation over the motor cortex enhances motor planning and functioning.

In contrast, greater beta activity is indicative of inhibited motor functioning. For instance, artificially increasing beta activity over the motor cortex via transcranial alternating-current stimulation (tACS) leads to slower reaction times in subsequent motor responses of the hands and fingers [12,13]. Additionally, Tzagarakis and colleagues [14] showed that uncertain future actions increased beta activity and slowed motor preparation, indicating that when participants are unable to plan for movement, beta activity increases.

One explanation for this relationship is that increased beta activity is associated with a state of motor maintenance in the sensorimotor cortex [11]. However, decreased beta activity “unlocks” the neurons in motor cortical pathways to facilitate motor actions by enhancing the excitability of corticospinal motor neurons [1,15]. Decreased beta patterns are also thought to share a neural network with levels of dopamine in subcortical regions. For instance, Parkinson’s patients who commonly have depleted dopamine levels have high levels of beta activation over motor regions. However, this effect is attenuated in those same patients when subcortical structures are stimulated to increase dopamine levels [16,17]. A connection between beta activation and dopamine also suggests that beta activity shares pathways of motivated motor preparation (e.g., reward predictions) [18,19].

### 1.2. Beta Activation and Motivation

When individuals experience states high in motivational intensity (strength), they exhibit greater cortical motor-action preparation [20]. States high in motivational intensity (e.g., for extrinsic rewards) are associated with enhancing cortical motor-action preparation that facilitates the acquisition of goals or resources [21,22,23]. The motivational intensity described in this literature, however, refers to motivational strength stemming from behaviors that must be exerted to obtain goals or resources [24]. This is different from motivational intensity comprised of both actual motivated behaviors, but also potential motivation described in earlier models [25]. Examining actual motivated behavior can be useful for addressing gaps in motivation and effort literature. Namely, where these motivation models explain some but not other behavioral effects, such as excesses in effort beyond difficulty demands [i.e., through cardiovascular physiology work by Gendolla and colleagues; see 43]. Specifically, since potential motivation is not necessarily indicative of need for engaging in a behavior [26], there is strong importance in examining how some of these diverging effects in psychophysiological research interact with cognitive processing, such as effect and motivation [27]. For example, research suggests beta activation decreases prior to reward trials relative to no-reward trials requiring the same performance-based actions [2]. Similar research using reward to facilitate motivation suggests beta activation is sensitive to motivated behavior, especially prior to motor movement [3]. Moreover, motivation for greater reward magnitudes not only decreases beta activation even further but can also mediate the length of physical breaks taken when those greater incentives are available [28].

Although beta activation is predominantly studied using strong extrinsic motivators (i.e., money), evidence suggests its sensitivity for intrinsically oriented motivators as well. For instance, when prompting individuals with the perceived expectancy of difficult and easy tasks based on social norms, beta activation decreased for difficult trials relative to easy trials even though actual task difficulty was not altered [29]. This is also in line with prior work suggesting the “gating role” of beta activity is further modulated beyond just the deactivation necessary to enable motor actions, especially since planned and often motivated motor actions are thought to also be responsible for decreased beta activation [1,30,31].

Lastly, individual differences in beta activation (i.e., unstimulated brain activity at rest) are also linked with individual differences in motivation. Decreased beta activity at rest was related for individuals who also reported greater behavioral activation system (BAS) [32] sensitivity [4]. Similarly, decreased beta activation was also related to another neural correlate of motivation (greater left frontal alpha activation) in individuals with high BAS traits, further emphasizing the link to motivation [33]. Altogether, these findings suggest the connection between beta activation over motor cortical regions is strongly linked to motor movements, but especially when these relate to motivation [2,3,21,29].

### 1.3. Beta, Motor Functioning, and Motivation

Past work has investigated the impact of motivation on decreased beta activity in preparation for a task [2,3,29], but little work has investigated how motivation influences beta activity during task performance. One reason for this is that past studies manipulating motivation used fast reaction time tasks that did not allow beta to be measured during the task. For instance, Pfurtscheller et al. [34] analyzed beta activity before and after movements, which on average ranged from 138 to 788 milliseconds (ms) [14,35].

Prior studies have also often used time-based goals rather than performance-based goals. For instance, instructing participants to move a finger for one to two seconds at a time revealed that beta activity decreased prior to movement, recovered during the first half of the movement, then decreased toward the end of the movement [36]. However, several of these tasks often use a relatively short time frame that prevents beta activation from being tracked over several seconds’ worth of motivated motor movement [9,37]. When assessing cortical beta activation in performance-based laboratory experiments, beta activation patterns have shown sensitivity to the degree of motivational intensity, but only prior to task demands [2,3,29].

In addition to the presence of a goal, information about goal pursuit progress can also impact motivation. Control theory of self-regulation [38] argues that feedback of progress provides information about how far one is from a desired goal and impacts subsequent motivated behavior. Therefore, if you are farther from the goal than you would like to be, you are likely to alter your behavior to increase the rate at which you approach the goal. If you are closer to the goal than is necessary (i.e., you are working harder than you need to), then you are likely to slow the rate at which you approach your goal. Essentially, if you are doing well, you might back off on your efforts. Threadgill [39] and Thürmer et al. [40] exhibited this “coasting” effect when giving participants accuracy and speed goals on a task and then presented with feedback after half of the trials were completed. When feedback suggested they were performing better than they needed to for their goal, they reduced their efforts and performed worse in the second half of the trials. This suggests feedback indicating near completion of a goal could lead to reduced effort and motivation during goal pursuit. Therefore, a reduction in the motivated effort should lead to a decrease in cortical motor-action preparation (i.e., an increase in beta activity).

### 1.4. The Present Study

In the present study, we measured beta activity over the motor cortex during an effort-modifying task. Participants engaged in a motor task requiring key presses on a keyboard. On each trial, participants could choose to perform a hard trial for more points or an easy trial for fewer points. Trials varied on the number of points that could be earned and the likelihood of earning points if the task was successfully completed.

With this manipulation, we sought to examine the impact of trial difficulty on beta activity in the motor cortex, as well as the relationship between preparatory beta activity and task performance with actual varying difficulty levels. It was hypothesized that motor beta activity would decrease during early preparatory periods for hard trials relative to easy trials. It was also expected that decreased preparatory beta activation would be associated with better performance on the task overall (i.e., greater total point values, greater success rate, and faster trial durations). Additionally, if trial point values and the probability of receiving points values enhance motivational intensity, then greater point values for hard trials and a higher probability of earning points should decrease beta activity. Since participants received feedback about how close they were to goal attainment via a progress bar, it was also hypothesized that beta activation would be higher toward the end of hard trials relative to the beginning of hard trials, as anticipation of future effort expenditure decreased.

## 2. Methods

### 2.1. Participants

Fifty-one (*n* = 51) introductory psychology students participated in exchange for partial course credit. To participate in the study, participants had to be at least 18 years old and provide informed consent. Because handedness can influence hemispheric cortical functioning [41], all participants in this study were also right-handed. Participant handedness was assessed using a 13-item checklist asking which hand (right, left, or both) they use to perform day-to-day tasks (e.g., writing, use scissors) [42]. Like in previous studies examining beta activation, participants were considered to be right-handed if they performed no more than one item with their left hand [2,3,4,29].

### 2.2. Procedures

Following informed consent, the EEG cap was applied to their scalp. Participants were then prompted to complete an effort expenditure for rewards task (EEfRT) [43]. Before starting the task, participants were told they should try to earn as many points as possible by completing as many trials as they could in 20 min. To incentivize participants to earn points, participants were told points would be exchanged for candy at the end of the experiment. Participants were then given instructions through the computer. Participants completed four practice trials to become familiar with the task.

**Figure 1:**
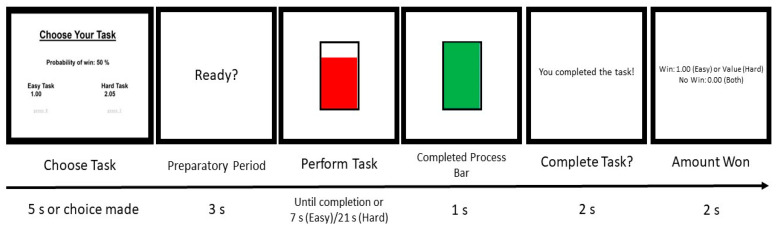
Stimuli presented to participants. The first screen prompts participants to choose to complete either an easy or hard trial based on the points that can be won and the likelihood of winning points given trial success. The second screen is presented for 3000 ms and allows participants to prepare for task execution. The third screen is presented during task execution; the red bar grows each time the participant presses the spacebar. The fourth screen is presented if the participant completes the desired number of spacebar presses successfully within the allotted time. The fifth screen informs participants of whether they successfully completed the trial. If the trial was completed successfully, the sixth screen informs participants of whether points were won (based on the probability of winning), as well as the quantity of points won.

At the start of each trial, participants were given the choice between performing an easy trial and performing a hard trial (see Figure 1). For the easy trials, participants had to click the spacebar 30 times in 7 s using the index finger of their dominant (right) hand. For the hard trials, participants had to click the spacebar 100 times in 21 s with the little finger on their non-dominant (left) hand.

Trial type also varied in potential points. All easy trials were worth 1 point. On the other hand, hard trials were worth between 1.24 and 4.30 points. To make sure participants earned money throughout the entire task (e.g., instead of choosing to earn all points at the beginning), task earning was kept consistent by informing participants that hard trials would take approximately twice as long as easy trials and that successfully completing a trial would not always yield the points that were chosen. To better inform them which trials would be more likely to lead to points, they were told that they either had a 12%, 50%, or 88% chance of actually receiving points if they successfully completed the trial [43]. If participants did not make a choice of trial type within five seconds, they were randomly assigned to a trial type.

After choosing whether to complete the easy or hard trial, participants then saw a screen that said, “Ready?” for 3000 ms, indicating the beginning of the preparatory period for the chosen trial. Participants then had 7 s or 21 s to complete the easy trial or hard trial, respectively. During this time, a vertical bar appeared on the screen that filled up as participants came closer to completing the chosen trial (i.e., their chosen goal).

After pressing the spacebar the required number of times or when the time limit was reached (whichever came first), a message stating whether the trial was completed appeared on the screen for 2000 ms. If participants successfully completed the trial, the phrase “You completed the task!” appeared. Otherwise, the phrase “Failure to complete” appeared. If the trial was not successfully completed, participants simply continued to the next trial. If participants successfully completed the trial, they were then told whether or not they had actually won points on the trial. This remained on the screen for 2000 ms. Participants then continued to the next trial. The EEfRT task was administered using Inquisit Software [44].

Task performance was measured across several behavioral variables. The number of points participants earned throughout the 20-min task was summed into a “total points” score. The “number of easy trials” completed was defined as the total number of easy trials completed in the 20-min task. The “percent easy” score was defined as the number of easy trials attempted divided by the total number of trials. The “easy success rate” score was defined as the number of successful, easy trials divided by the total number of easy trials. Success was not contingent on receiving points. “Easy task duration” was defined as the average amount of time spent on easy trials. Equivalent variables were also calculated for the hard trials.

### 2.3. EEG Assessment and Processing

Electroencephalography was recorded from 64 tin electrodes mounted in a stretch lycra Quick-Cap (Electro-Cap, Eaton, OH, USA) and referenced online to the left earlobe. A ground electrode was mounted midway between FPz and Fz (electrode positioning based on a 10–20 system). Data were then re-referenced offline to a linked-ears reference. Electrode impedances were kept under 5 kΩ. Signals were amplified with a Neuroscan SynAmps RT amplifier unit (El Paso, TX, USA), low-pass filtered at 100 Hz, high-pass filtered at 0.05 Hz, notch filtered at 60 Hz, and digitized at 500 Hz. Artifacts (e.g., horizontal eye movement and muscle) were removed by hand. Then, an automated regression-based eye movement correction was applied using site FP1 as the reference channel to correct vertical eye blinks [45], after which the data were visually inspected again to ensure proper correction.

During the 3000 ms preparatory period and throughout trial execution, 1024 ms epochs were extracted using a sinusoidal-shaped Hamming window to reduce spectral leakage (50% taper of distal ends) [46]. Consecutive epochs were overlapped by 50% to avoid data loss. Next, power values corresponding to beta (13–30 Hz) were extracted using a fast Fourier transformation. Data were then averaged across sites on the head corresponding to the motor cortex [37,47,48]. Analyses specifically examined beta activity at sites C1, C2, C3, C4, C5, C6, CP1, CP2, CP3, CP4, CP5, and CP6. For preparatory periods, participants had an average of 180 epochs for easy trials and 98 epochs for hard trials. During the task, participants had an average of 368 epochs for the easy trials, 186 epochs for the early phase of hard trials, 187 epochs for the middle phase of hard trials, and 146 epochs for the late phase of hard trials.

### 2.4. Statistical Analyses

Four total participants were excluded due to data loss, leaving 47 participants (mean age = 18.48, SD = 0.86, 34.04% female). Data were checked for outliers for all variables of interest (beta activity variables and behavioral variables). For each variable, a participant was excluded prior to analyses if they were >3 *SD*s from the mean for that variable (representing about <0.01% of a standard normal curve), well beyond the 25th and 75th percentiles that are typically considered as outliers by upper and lower fences of a data observations [49]. This practice is also consistent with motivation research examining beta activation in psychological contexts [2,29]. One participant was removed as an outlier from all analyses examining beta activity. One additional participant was removed as an outlier from all analyses examining beta activity during preparation for hard trials and during task execution. The other two participants experienced computer problems and did not have EEG recordings.

### 2.5. Beta Activation Analyses

To test for relative differences in beta activation during the preparatory period (i.e., during “Ready?”), a 2 (trial difficulty: easy vs. hard) × 3 (point likelihood: 12% vs. 50% vs. 88%) repeated-measures ANOVA was conducted to determine differences in beta activation between hard and easy and their respective point likelihood. Bivariate correlations were also examined between preparatory beta activation for hard trials, easy trials, total points, percent of easy trials, easy success rate, and easy trial duration. To further test the difference of these observed correlations, we used a Steiger’s Z-test [50,51]. To examine beta activation during the task (i.e., as participants were seeing the progress bar increase or trial timed out), and because hard trials had to last three times longer than easy trials [43], beta activity during hard trials was broken up into three equal segments (early, middle, and late). This would also ensure an equal time comparison between early beta activation for hard trials would be as long as the easy trial period. For this analysis, a one-way ANOVAs was conducted for early task beta between hard and easy trials. An additional one-way ANOVA was conducted only for hard trials to test whether beta activation is attenuated early on but increases as progress continues. Bivariate correlations were also conducted with the same behavioral measures of interest as the preparatory beta period.

## 3. Results

### 3.1. Preparatory Beta

A 2 (trial difficulty: easy vs. hard) × 3 (point likelihood: 12, 50, 88) repeated-measures ANOVA revealed only a main effect for trial difficulty, *F* (1,46) = 20.90, *p* < 0.001, *η_p_*^2^ = 0.31, where the preparatory period prior to hard trials (M = 0.61, SD = 0.33) decreased beta activation relative to easy trials (M = 0.70, SD = 0.36). There was no main effect of point likelihood (*p* > 0.11) and no interaction (*p* > 0.77). These results suggest there was greater cortical motor-action preparation when participants were anticipating hard trials relative to easy trials.

When further examining the link between preparatory beta activity and task performance, bivariate correlations revealed beta activation during the preparatory period of hard trials was negatively associated with behavioral outcomes reflecting enhanced task performance such as total points (*r* = −0.38, *p* < 0.01) and success rate (*r* = −0.40, *p* < 0.01). Preparatory beta activation for easy trials was significantly associated with total points (*r* = −0.32, *p* < 0.03), but not success rate (*r* = 0.05, *p* > 0.71) on those trials (see Table 1).

**Figure 2:**
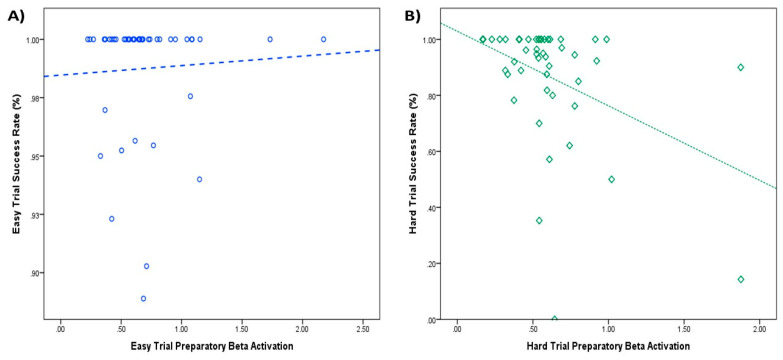
Preparatory beta activation and trial success rates. (**A**) Easy trial preparatory beta activation does not predict easy trial success rates (**blue**). (**B**) Hard trial preparatory beta activation predicts hard trial success rates (**green**). Lower beta activation scores represent greater cortical motor-action preparation. Higher trial success rates represent better performance.

To test whether one correlation was stronger than another between significant correlations for hard and easy trials, respectively, a Steiger’s Z-test [50,51] was conducted for total points and respective success rate correlations. This test revealed that the correlations for total points were not significantly different (*Z* = 1.14, *p* > 0.25). This seemed in line with the conceptual design of the task because both trials contributed to total points. However, there was a significant difference (*Z* = 2.34, *p* < 0.02) examining the difference in correlations between easy and hard preparatory beta activation and their respective trial success, indicating early beta activation and trial success were more strongly correlated for hard trials than for easy trials. To further test whether early beta activation predicted success rates in their respective trials, simple linear regression analyses were conducted to predict trial success based on early beta activation on respective trials. The regression equation was non-significant for easy preparatory beta predicting easy trial success, *F*(1,46) = 0.11, *p* > 0.73. However, the regression equation was significant for hard trials, *F*(1,46) = 6.36, *p* < 0.02, *R*^2^ = 0.35, where decreased beta activation predicted greater hard trial success (β = −0.22, *p* < 0.02; see Figure 2).

Altogether, these results suggest there was greater cortical motor preparation during preparatory periods for hard trials relative to easy trials and that greater cortical motor preparation was predicted better task performance in hard trials.

### 3.2. Task Beta

**Figure 3:**
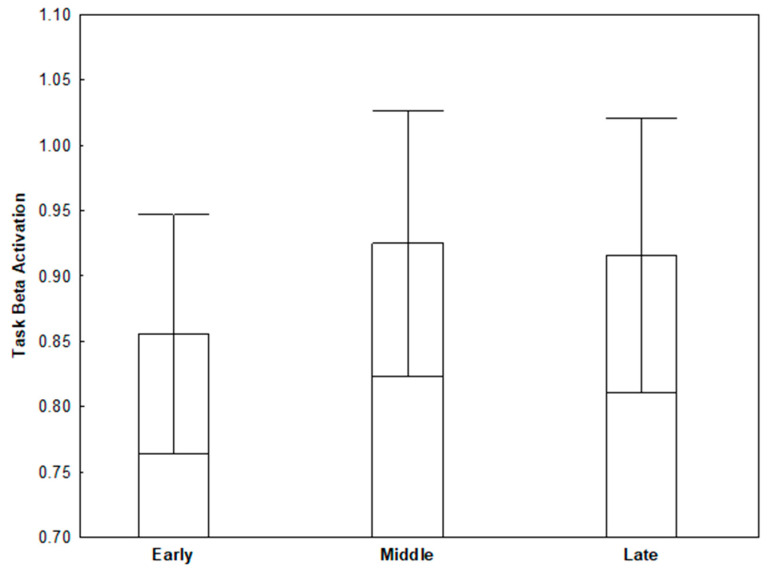
Task beta activation during hard trials depicting decreased beta activation for the early segment of the hard trials relative to the middle and late segments. Error bars indicate standard errors from the mean.

A repeated-measures ANOVA revealed decreased beta activity during the easy trials (*M* = 0.74, *SD* = 0.58) relative to the first third of hard trials (i.e., early phase of hard trials; *M* = 0.86, *SD* = 0.63), *F*(1, 46) = 16.02, *p* < 0.001, *η_p_*^2^ = 0.26. This suggests cortical motor-action preparation was no longer greater for hard trials once the start of the trials had begun, since greater motor preparation had already occurred during the preparatory phase. When examining beta activity during equal thirds of the longer hard trials, a one-way ANOVA revealed an omnibus trend, *F*(2, 92) = 2.22, *p* = 0.114, *η_p_*^2^ = 0.05. Follow-up analyses revealed decreased beta activity during the early phase (*M* = 0.86, *SD* = 0.63) relative to the middle phase (*M* = 0.93, *SD* = 0.70) of hard trials (*p* = 0.055, *d* = 0.27). The early phase of hard trials had marginally decreased beta activity relative to the late phase of hard trials (*M* = 0.92, *SD* = 0.72; *p* = 0.095, *d* = 0.20). Beta activation was not significantly different between middle and late phases of the hard trials (*p* > 0.79). This evidence suggests beta activation increased over the course of hard trial segments, indicating diminished cortical motor-action preparation as hard trials progressed (see Figure 3).

When further examining the link between task beta activity and task performance, bivariate correlations revealed beta activation was negatively associated with eventual trial success rates during the middle (*r* = −0.33, *p* < 0.05) and late (*r* = −0.32, *p* < 0.05) portions, while still trending in the same direction during early hard trials (*r* = −0.24, *p* = 0.10). This suggests that maintaining low beta activation throughout the hard trials was related to eventual trial success, a relationship not found with easy trials (*r* = 0.11, *p* > 0.45). This relationship was also similar, with total points gained as well (see Table 1). Steiger’s Z tests on correlation differences did not reveal any significant differences (*p*s > 0.17). To investigate the relationship of whether changes in beta within hard trials related to trial success, we created a difference score (early-late; lower scores indicated decreased beta in earlier compared to late segments) and correlated it with hard trial success, which was marginally significant (*r* = 0.26, *p* = 0.069). This suggests that the relationship between maintaining decreased beta throughout the trial was marginally correlated with better trial success, once again indicating the link between decreased beta activity and task performance.

## 4. Discussion

The relationship between beta activity in preparation for and during the execution of motor tasks and subsequent performance was primarily moderated by task difficulty. Beta activity is suppressed in preparation for a difficult motor task relative to an easy motor task. Explicitly denoting that the trials differed in difficulty suppressed beta activation in the motor cortical areas relative to an objectively easier task. This suggests that when preparing for a more challenging task, people exhibited greater cortical motor-action preparation, a reflection of enhanced motivational intensity [2,3,29]. Together, these results are consistent with the growing body of work investigating how motivation and task difficulty influence cortical motor preparation [11,16], but specifically that task difficulty (e.g., greater task demands) may enhance motivational intensity to perform [29].

During the hard trial performance, there was a relative beta activation increase from the early to the middle and late phases of hard trials. Perhaps as participants came closer to achieving their goal (through repetitive movement when viewing the progress bar on the screen), their motor movements became less planful following each key press. This is in line with prior research suggesting cortical beta activation is primarily tied to purposeful and planned action [4,11,14].

Beta activity during easy trials was predominantly unrelated to success rates in those trials. In contrast, decreased beta activation in hard trials was associated with task performance during the preparatory phase and task phase. Moreover, this relationship is maintained throughout the early, middle, and late phases of hard trials. Correlational results further highlight the link between motivated motor movement processing and contexts demanding greater motivational intensity via varying difficulty levels.

Importantly, a future direction with this body of work should perhaps examine the link between motivation and anxiety attenuation for laboratory tasks varying in difficulty by examining cortical beta suppression. Because beta activation is thought to represent a “gating role” in enabling future cell firing for motivated goal pursuit [12,52], it represents a more immediate indicator of motivational intensity. Perhaps examining a potential link between motivational intensity, task difficulty, and levels of anxiety toward task performance may elucidate some of the current research on anxiety. Specifically, some debate exists as to the effects of anxiety in performance-based studies [53], where the linear relationship between anxiety and motivation has come under some scrutiny. This body of research suggests that some levels of anxiety may be catalysts for motivation up to a point [8,53], and that expectations of success, motivation, and anxiety interact with each other across various learning or task outcomes [54,55]. Importantly, these results revealed no concrete adverse behavioral or neurological attenuation of cortical preparation for the impetus to act as a result of a more demanding task [7,8]. This is in line with prior work suggesting that effort demands may enhance motivational strength for an activity as a result of heightened task demands [6] and perceived difficulty [29].

Moreover, future studies could perhaps examine the link between beta activation and salience network (SN) for rewards. Because salience network activity is linked with activity in regions of reward anticipation (dACC, inula, and pupillometry) [56] and insular activity tied to attentional control for relevant stimuli to guide behavior [57,58], this network may also be tied to preparatory beta activation when motivation is relatively higher for an activity or resource. Prior research on SN activity and EEG frequency has predominantly focused on alpha band frequency (8–12 Hz) fluctuations [59] and how these may be affected by anxious traits or diagnosis [60]. However, recent studies have found a link between beta activation and SN activity in individuals experiencing pain [61]. Importantly, individuals experiencing comorbidity with pain appear to have decreased beta activation as it relates to salience (SN activity) to potential pain due to movement [62]. In some cases, neurofeedback training increasing beta activation in neural networks has attenuated activity within these networks [63], yet more research on this potential link between beta frequency (and sometimes alpha) is needed as this effect has not always been observed [64].

While the present study has a number of strengths, there are limitations that should be noted. First, the inclusion of an uncertainty of earning points likely impacted results in unanticipated ways. It likely complicated the decision-making process for participants, possibly resulting in the unexpected lack of interaction with trial difficulty on beta activation. Second, the reminder in the instructions that choosing more hard trials early on could cause participants to lose out on high-point trials later [43] may have skewed results. For instance, highly motivated individuals who might otherwise choose many hard trials could have been persuaded away from that tendency. Further, while point values offered for hard trials were large enough to elicit high motivation overall, point differences within hard trials may not have been large enough to alter beta activity. This would be in line with Mirabella’s [65] results suggesting that motor preparation is particularly directed toward high-value goals: all hard point values may have been considered “high-value” since easy trials were always worth only one point.

Despite these limitations, the present study is one of the first to investigate motivation both in preparation for movement and execution of that movement on beta activity over the motor cortex across varying degrees of perceived difficulty. Past studies have investigated beta activity before and after movement [14,34,35], while many more have investigated movement itself without any goal or reward for doing so [9,36,37]. The present study indicates that while participants were motivated to complete a difficult task, beta activity increased throughout the course of hard trials. This is in line with Carver and Scheier’s [38] control theory of self-regulation: as participants became ever closer to fulfilling their goal, motor preparation decreased (i.e., beta activity increased). As the progress bar filled, participants could see that they had fewer button presses to make, and the need to prepare for movement declined.

Overall, the results of the present study provide insight into how beta activation over the motor cortex interacts with task difficulty to facilitate readiness and effort expenditure. A hard task elicited greater relative cortical motor preparation than an easier task. Additionally, motor preparation declines as participants progress through hard trials. Taken together, the present results further elucidate the effects of motivational intensity between actual easy and hard task difficulty on cortical activation that facilitates our impetus to act and engage in goal pursuit.

## Figures and Tables

**Table 1:** Correlations between beta activation and behavioral outcome variables during easy and hard trials. Beta activation is shown separately for the preparatory phase and the task execution phase. Due to the relatively short length of easy trials, the task execution phase is listed as one segment. The task execution phase of hard trials is divided into thirds: early, middle, and late. The difference between the late and early thirds is also represented for hard trials.

Easy Trials	Total Points	Percent Easy	Easy Success Rate	Easy Task Duration
Preparatory beta	−0.32 **	0.15	0.05	0.28 ^†^
Task beta	−0.30 **	0.11	0.11	0.17
Hard Trials	Total points	Percent hard	Hard success rate	Hard task duration
Preparatory beta	−0.38 *	−0.04	−0.40 *	0.30 **
Early Task Beta	−0.25 ^†^	−0.10	−0.24 ^†^	0.16
Middle Task Beta	−0.31 **	−0.18	−0.33 **	0.16
Late Task Beta	−0.28 ^†^	−0.18	−0.32 **	0.22
Task Beta: (Late–Early)	−0.14	−0.21	−0.27 ^†^	0.18

* *p* < 0.01, ** *p* < 0.05, ^†^ *p* < 0.10.

## Data Availability

Data can be made available upon request.

## References

[1] Neuper C., Pfurtscheller G. (2001). Event-related cortical rhythms: Frequency-specific features and functional correlates. Int. J. Psychophysiol..

[2] Gable P.A., Threadgill A.H., Adams D.L. (2016). Neural activity underlying motor-action preparation and cognitive narrowing in approach-motivated goal states. Cogn. Affect. Behav. Neurosci..

[3] Meadows C.C., Gable P.A., Lohse K.R., Miller M.W. (2016). Motivation and motor cortical activity can independently affect motor performance. Neuroscience.

[4] Threadgill A.H., Gable P.A. (2018). Resting beta activation and trait motivation: Neurophysiological markers of motivated motor-action preparation. Int. J. Psychophysiol..

[5] Kurzban R. (2016). The sense of effort. Curr. Opin. Psychol..

[6] Inzlicht M., Shenhav A., Olivola C. (2018). The Effort Paradox: Effort Is Both Costly and Valued. Trends Cogn. Sci..

[7] Caviola S., Carey E., Mammarella I.C., Szucs D. (2017). Stress, Time Pressure, Strategy Selection and Math Anxiety in Mathematics: A Review of the Literature. Front. Psychol..

[8] Luttenberger S., Wimmer S., Paechter M. (2018). Spotlight on math anxiety. Psychol. Res. Behav. Manag..

[9] Yuan H., Liu T., Szarkowski R., Rios C., Ashe J., He B. (2010). Negative covariation between task-related responses in alpha/beta-band activity and BOLD in human sensorimotor cortex: An EEG and fMRI study of motor imagery and movements. NeuroImage.

[10] Van Wijk B.C.M., Daffertshofer A., Roach N., Praamstra P. (2009). A role of beta oscillatory synchrony in biasing response competition?. Cereb. Cortex.

[11] Engel A.K., Fries P. (2010). Beta-band oscillations—Signalling the status quo?. Curr. Opin. Neurobiol..

[12] Pogosyan A., Gaynor L.D., Eusebio A., Brown P. (2009). Boosting Cortical Activity at Beta-Band Frequencies Slows Movement in Humans. Curr. Biol..

[13] Wach C., Krause V., Moliadze V., Paulus W., Schnitzler A., Pollok B. (2013). Effects of 10 Hz and 20 Hz transcranial alternating current stimulation (tACS) on otor functions and motor cortical excitability. Behav. Brain Res..

[14] Tzagarakis C., Ince N.F., Leuthold A.C., Pellizzer G. (2010). Beta-Band Activity during Motor Planning Reflects Response Uncertainty. J. Neurosci..

[15] Chen R., Yaseen Z., Cohen L.G., Hallett M. (1998). Time course of corticospinal excitability in reaction time and self-paced movements. Ann. Neurol..

[16] Jenkinson N., Brown P. (2011). New insights into the relationship between dopamine, beta oscillations and motor function. Trends Neurosci..

[17] Kuhn A.A., Kempf F., Brucke C., Gaynor Doyle L., Martinez-Torres I., Brown P. (2008). High-frequency stimulation of the subthalamic nucleus suppresses oscillatory beta activity in patients with Parkinson’s disease in parallel with improvement in motor performance. J. Neurosci..

[18] Schulz W. (2016). Dopamine reward prediction error coding. Dialogues Clin. Neurosci..

[19] Tobler P.N., Fiorillo C.D., Schultz W. (2005). Adaptive Coding of Reward Value by Dopamine Neurons. Science.

[20] Doyle L.M., Kühn A.A., Hariz M., Kupsch A., Schneider G.H., Brown P. (2005). Levadopa-induced modulation of subthalamic beta oscillations during self-paced movements in patients with Parkinson’s Disease. Eur. J. Neurosci..

[21] Babiloni C., Del Percio C., Vecchio F., Sebastiano F., Di Gennaro G., Quarato P.P., Morace R., Pavone L., Soricelli A., Noce G. (2016). Alpha, beta, and gamma electrocorticographic rhythms in somatosensory, motor, premotor, and prefrontal cortical areas differ in movement execution and observation in humans. Clin. Neurophysiol..

[22] Cunnington R., Windischberger C., Deecke L., Moser E. (2002). The preparation and execution of self-initiated and externally-triggered movement: A study of event-related fMRI. NeuroImage.

[23] Sanes J.N., Donoghue J.P. (1993). Oscillations in local field potentials of the primate motor cortex during voluntary movement. Proc. Natl. Acad. Sci. USA.

[24] Gable P.A., Harmon-Jones E. (2010). The motivational dimensional model of affect: Implications for breadth of attention, memory, and cognitive categorization. Cogn. Emot..

[25] Brehm J.W., Self E.A. (1989). The intensity of motivation. Ann. Rev. Psychol..

[26] Wright R.A., Gollwitzer P.M., Bargh J.A. (1996). Brehm’s theory of motivation as a model of effort and cardiovascular response. The Psychology of Action: Linking Cognition and Motivation to Behavior.

[27] Silvestrini N., Gendolla G. (2019). Affect and cognitive control: Insights from research on effort mobilization. Int. J. Psychophysiol..

[28] Meyniel F., Pessiglione M. (2014). Better get back to work: A role for motor beta desynchronization in incentive motivation. J. Neurosci..

[29] Wilhelm R.A., Miller M.W., Gable P.A. (2019). Neural and Attentional Correlates of Intrinsic Motivation Resulting from Social Performance Expectancy. Neuroscience.

[30] Salenius S., Schnitzler A., Salmelin R., Jousmäki V., Hari R. (1997). Modulation of Human Cortical Rolandic Rhythms during Natural Sensorimotor Tasks. NeuroImage.

[31] Schnitzler A., Salenius S., Salmelin R., Jousmäki V., Hari R. (1997). Involvement of Primary Motor Cortex in Motor Imagery: A Neuromagnetic Study. NeuroImage.

[32] Carver C.S., White T.L. (1994). Behavioral inhibition, behavioral activation, and affective responses to impending reward and punishment: The BIS/BAS scales. J. Personal. Soc. Psychol..

[33] Wendel C.J., Wilhelm R.A., Gable P.A. (2021). Individual Differences in Motivation and Impulsivity Link Resting Frontal Alpha Asymmetry and Motor Beta Activation. Biol. Psychol..

[34] Pfurtscheller G., Zalaudek K., Neuper C. (1998). Event-related beta synchronization after wrist, finger and thumb movement. Electroencephalogr. Clin. Neurophysiol. Mot. Control..

[35] Zhang Y., Chen Y., Bressler S., Ding M. (2008). Response preparation and inhibition: The role of the cortical sensorimotor beta rhythm. Neuroscience.

[36] Stancák A., Pfurtscheller G. (1996). Event-related desynchronisation of central beta-rhythms during brisk and slow self-paced finger movements of dominant and nondominant hand. Cogn. Brain Res..

[37] McFarland D.J., Miner L.A., Vaughan T.M., Wolpaw J. (2000). Mu and Beta Rhythm Topographies During Motor Imagery and Actual Movements. Brain Topogr..

[38] Carver C.S., Scheier M.F. (1982). Control theory: A useful conceptual framework for personality–social, clinical, and health psychology. Psychol. Bull..

[39] Threadgill A.H. (2019). From Preparation to Assessment: Exploring the Neural Substrates of Approach-Motivated Goal Pursuit. Doctoral Dissertation.

[40] Thürmer J.L., Scheier M.F., Carver C.S. (2020). On the mechanics of goal striving: Experimental evidence of coasting and shifting. Motiv. Sci..

[41] Heller W., Levy J. (1981). Perception and expression of emotion in right-handers and left-handers. Neuropsychologia.

[42] Chapman L.J., Chapman J.P. (1987). The measurement of handedness. Brain Cogn..

[43] Treadway M.T., Buckholtz J.W., Schwartzman A.N., Lambert W.E., Zald D.H. (2009). Worth the ‘EEfRT’? The Effort Expenditure for Rewards Task as an Objective Measure of Motivation and Anhedonia. PLoS ONE.

[44] (2015). Inquisit 4.0.10. [Computer Software]. http://www.millisecond.com.

[45] Semlitsch H.V., Anderer P., Schuster P., Presslich O. (1986). A Solution for Reliable and Valid Reduction of Ocular Artifacts, Applied to the P300 ERP. Psychophysiology.

[46] Davidson R.J., Jackson D.C., Larson C.L., Cacioppo J.T., Tassinary L.G., Berntson G.G. (2000). Human electroencephalography. Handbook of Psychophysiology.

[47] Muthukumaraswamy S.D., Johnson B.W., McNair N.A. (2004). Mu rhythm modulation during observation of an object-directed grasp. Cogn. Brain Res..

[48] Pfurtscheller G., Neuper C., Brunner C., da Silva F.L. (2005). Beta rebound after different types of motor imagery in man. Neurosci. Lett..

[49] Kwak S.K., Kim J.H. (2017). Statistical data representation: Management of missing values and outliers. Korean J. Anesthesiol..

[50] Lee I.A., Preacher K.J. (2013). Calculation for the Test of the Difference between Two Dependent Correlations with One Variable in Common [Computer Software]. http://quantpsy.org.

[51] Steiger J.H. (1980). Tests for comparing elements of a correlation matrix. Psychol. Bull..

[52] Chen C.C., Litvak V., Gilbertson T., Kühn A., Lu C.S., Lee S.T., Tsai C.H., Tisch S., Limousin P., Hariz M. (2007). Excessive synchronization of basal ganglia neurons at 20 Hz slows movement in Parkinson’s disease. Exp. Neurol..

[53] Wang Z., Lukowski S.L., Hart S.A., Lyons I.M., Thompson L.A., Kovas Y., Mazzocco M.M.M., Plomin R., Petrill S.A. (2015). Is Math Anxiety Always Bad for Math Learning? The Role of Math Motivation. Psychol. Sci..

[54] Paechter MMacher D., Martskvishvili K., Wimmer S., Papousek I. (2017). Mathematics anxiety and statistics anxiety. Shared but also unshared components and antagonistic contributions to performance in statistics. Front. Psychol. Educ. Psychol..

[55] Skaalvik E.M. (2018). Mathematics anxiety and coping strategies among middle school students: Relations with students’ achievement goal orientations and level of performance. Soc. Psychol. Educ..

[56] Schneider M., Leuchs L., Czisch M., Sämann P.G., Spoormaker V.I. (2018). Disentangling reward anticipation with simultaneous pupillometry/fMRI. NeuroImage.

[57] Menon V., Uddin L.Q. (2010). Saliency, switching, attention and control: A network model of insula function. Brain Struct. Funct..

[58] Seeley W.W., Menon V., Schatzberg A.F., Keller J., Glover G.H., Kenna H., Reiss A.L., Greicius M.D. (2007). Dissociable Intrinsic Connectivity Networks for Salience Processing and Executive Control. J. Neurosci..

[59] Ros T., Theberge J., Frewen P.A., Kluetsch R., Densmore M., Calhoun V.D., Lanius R.A. (2013). Mind over chatter: Plastic up-regulation of the fMRI salience network directly after EEG neurofeedback. NeuroImage.

[60] Massullo C., Carbone G.A., Farina B., Panno A., Capriotti C., Giacchini M., Machado S., Budde H., Murillo-Rodríguez E., Imperatori C. (2020). Dysregulated brain salience within a triple network model in high trait anxiety individuals: A pilot EEG functional connectivity study. Int. J. Psychophysiol..

[61] Wang W., Ho RL M., Gatto B., van der Veen S.M., Underation M.K., Thomas J.S., Antony A.B., Coomes S.A. (2021). Cortical dynamics of movement-evoked pain in chronic low back pain. J. Physiol..

[62] Kim J.A., Bosma R.L., Hemington K.S., Rogachov A., Osborne N.R., Cheng J.C., Oh J., Crawley A.P., Dunkley B.T., Davis K.D. (2019). Neuropathic pain and pain interference are linked to alpha-band slowing and reduced beta-band magnetoencephalography activity within the dynamic pain connectome in patients with multiple sclerosis. Pain.

[63] Shtark M.B., Kozlova L.I., Bezmaternykh D.D., Mel’Nikov M.Y., Savelov A., Sokhadze E.M. (2018). Neuroimaging Study of Alpha and Beta EEG Biofeedback Effects on Neural Networks. Appl. Psychophysiol. Biofeedback.

[64] Kisler L.B., Kim J.A., Hemington K.S., Rogachov A., Cheng J.C., Bosma R.L., Osborne N.R., Dunkley B.T., Inman R.D., Davis K.D. (2020). Abnormal alpha band power in the dynamic pain connectome is a marker of chronic pain with a neuropathic component. NeuroImage Clin..

[65] Mirabella G. (2014). Should I stay or should I go? Conceptual underpinnings of goal-directed actions. Front. Syst. Neurosci..

